# Myasthenia gravis and paroxysmal nocturnal hemoglobinuria after thymectomy: A rare association

**DOI:** 10.1002/jha2.244

**Published:** 2021-09-29

**Authors:** Jean Galtier, Fanny Duval, Irène Machelart, Carine Greib, Estibaliz Lazaro, Jean‐Luc Pellegrin, Jean‐François Viallard, Regis Peffault de la Tour, Etienne Rivière

**Affiliations:** ^1^ Hematology Department Haut‐Leveque Hospital University Hospital Center of Bordeaux Pessac France; ^2^ Neurology Department Pellegrin Hospital University Hospital Center of Bordeaux Bordeaux France; ^3^ Internal Medicine and Infectious Diseases Haut‐Leveque Hospital University Hospital Center of Bordeaux Pessac France; ^4^ INSERM U1034 Pessac Cedex France; ^5^ Service d'Hématologie Greffe Centre de référence des aplasies médullaires acquises et constitutionnelles Hôpital Saint Louis AP‐HP Paris France

**Keywords:** aplastic anemia, autoimmune disease, immunohematology

## Abstract

Paroxysmal nocturnal hemoglobinuria (PNH) is a very rare clonal autoimmune disease manifesting with hemolysis, thrombosis, or bone marrow failure. We present an atypical association of myasthenia gravis, aplastic anemia, and PNH occurring years after thymectomy. While this association might be extremely rare, it may not be coincidental as there is a common pathophysiology between PNH and aplastic anemia, with the latter reported in several thymoma/thymectomy cases. Eculizumab was introduced with good efficacy and without safety concern in our patient, leading to long‐term control of PNH without worsening of myasthenia gravis.

We report the case of a 56‐year‐old man referred to our center for worsening pancytopenia. He had been diagnosed with myasthenia gravis (MG, oropharyngeal type) associated with thymic hyperplasia in 1992 and was treated by thymectomy, steroids, and anticholinesterase medication. Anti‐AChR antibodies were detectable at that time. Despite thymectomy, steroids and anticholinesterase were still necessary, with occasional flares treated with methylprednisolone. Of note, all the blood counts were unremarkable until 2011.

In 2011, he described chronic asthenia with no abdominal pain, thrombotic events, or erectile dysfunction. His biological tests demonstrated neutropenia (0·8 × 10^9^/L), thrombocytopenia (40 × 10^9^/L), incompletely compensated hemolytic anemia (hemoglobin at 10 g/dl, reticulocytes 50 × 10^9^/L) with increased lactate dehydrogenase (LDH) (four‐fold the normal value), and undetectable haptoglobin. The direct agglutination tests were negative. Bone marrow biopsy revealed slightly decreased cellularity with dyserythropoiesis and hypoplasia in the neutrophil lineage. Whole body CT scan was normal and serum parameters indicative of autoimmune disease, such as antinuclear antibodies, anti‐DNA antibodies, and rheumatoid factor, were not detected. Lastly, a paroxysmal nocturnal hemoglobinuria (PNH) clone was detected, with a size of 6% of granulocytes (2% of erythrocytes) in 2011, that further increased to 58% in granulocytes (14% of erythrocytes) in 2012.

The eventual diagnosis was an association of nonsevere aplastic anemia (AA) with PNH. As there were no severe AA criteria, no need for transfusion, and no thrombotic events, the patient was closely monitored until November 2013 when hemolysis worsened, requiring a red cell transfusion. PNH clone size was stable (59% of granulocytes and 15% of erythrocytes). The complement protein 5 (C5) inhibitor eculizumab was started in January 2014 allowing for a rapid LDH decrease (Figure 1). In the first 6 months, two red blood cell transfusions were required, most likely due to the underlying AA. At the end of 2014, reticulocyte, neutrophil, and platelet counts spontaneously rose (Figure 2). Of note, no clonal evolution toward myelodysplastic syndrome occurred, with three bone marrow aspirations performed between 2014 and 2018, which did not show either myelodysplasia or karyotype abnormalities. The patient became transfusion‐independent and importantly, his MG remained relatively quiescent after eculizumab treatment began, with a concomitant daily treatment of 20 mg prednisone and anticholinesterase. On four occasions between 2014 and 2018, he received a bolus injection of 1 g methylprednisolone for isolated episodes of diplopia.

**FIGURE 1:**
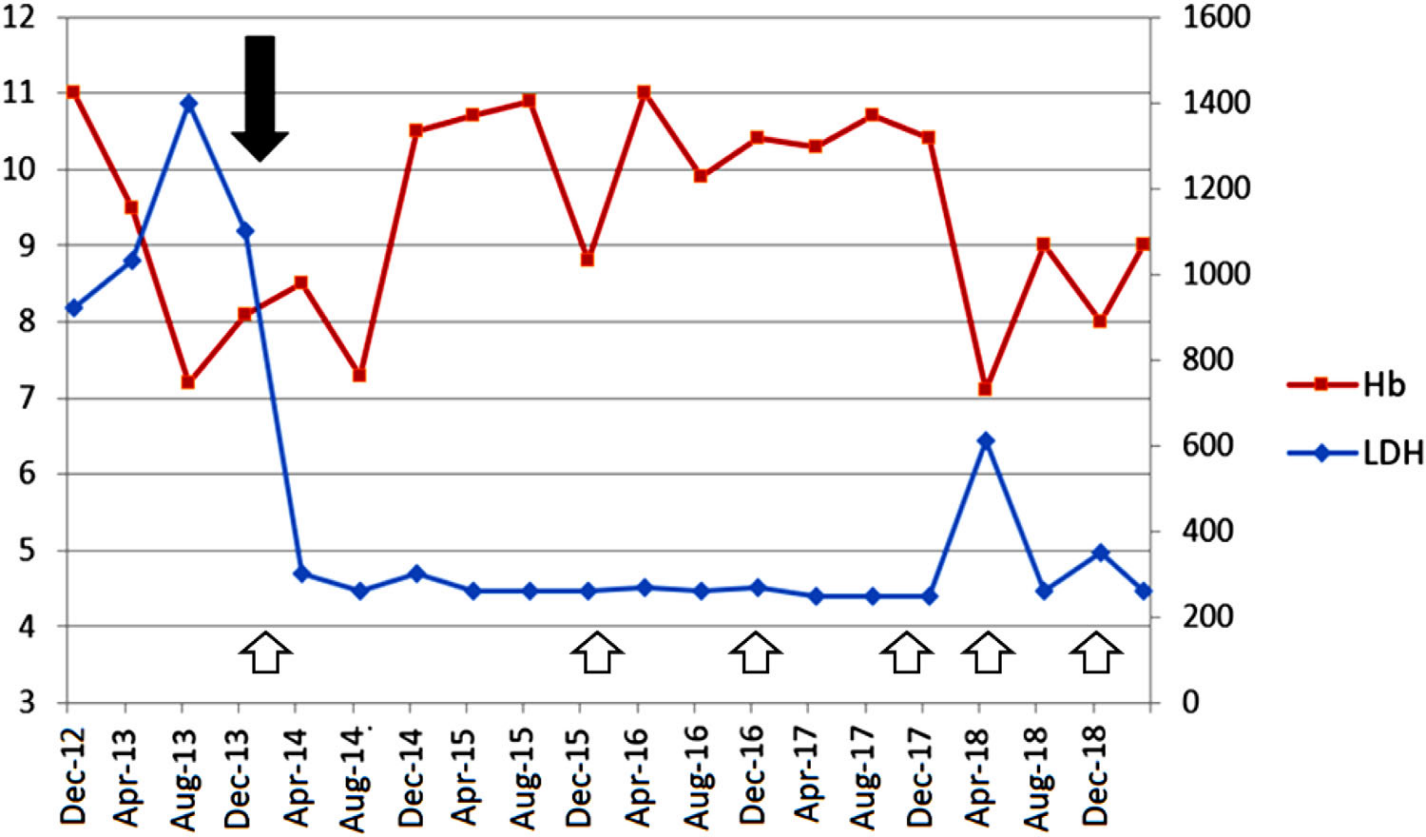
Hemoglobin (Hb) and LDH values in g/dl and UI/L, respectively. Arroweheads: infectious episodes. Black arrow: introduction of eculizumab

**FIGURE 2:**
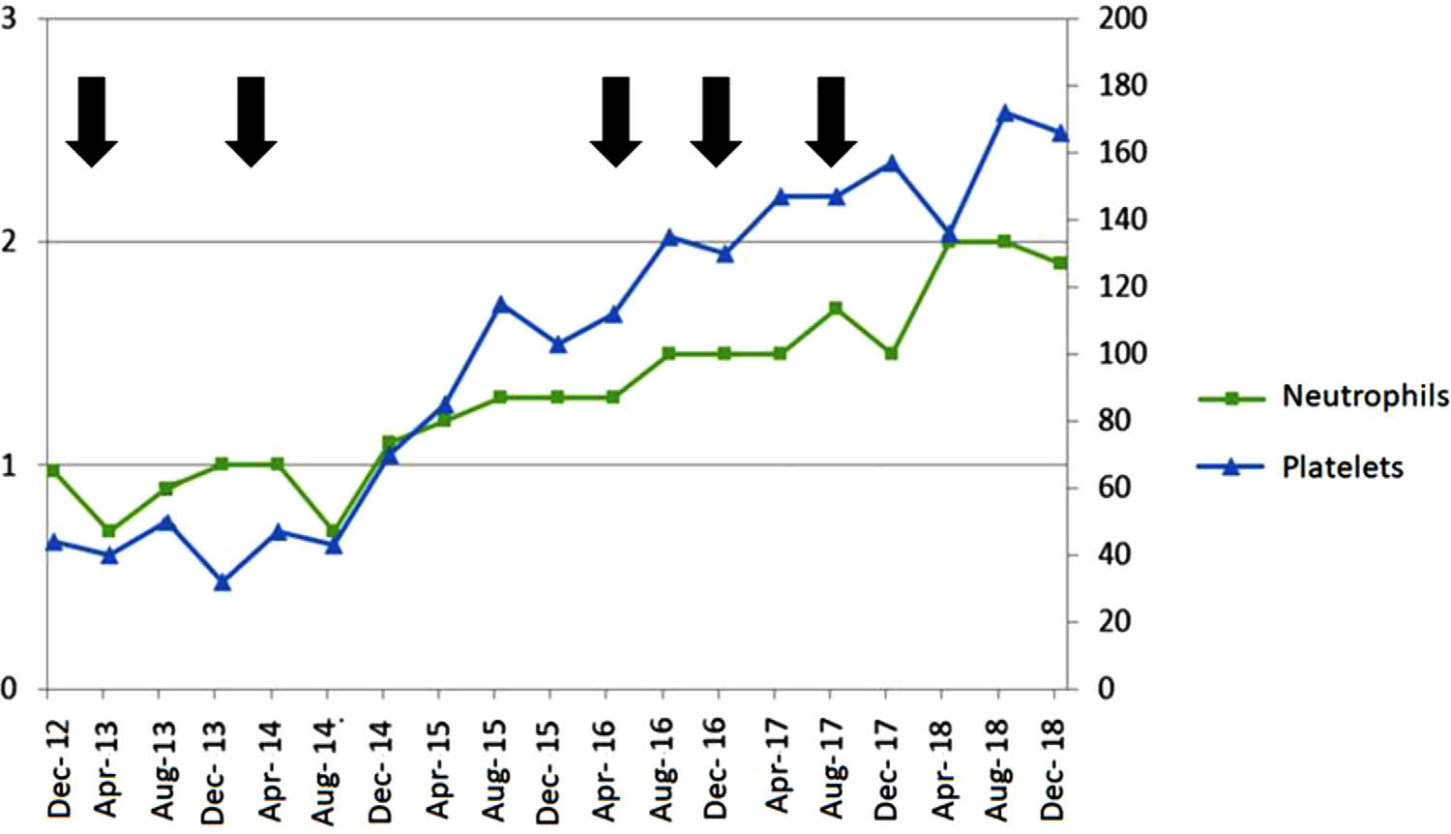
Evolution of neutrophil and platelet counts (x 10^9^/L). *Arrows: steroid bolus for myasthenia gravis flares*

PNH is a very rare clonal autoimmune disease manifesting with hemolysis, thrombosis, or bone marrow failure. It is explained by acquired mutations in the phosphatidylinositol N‐acetylglucosaminyltransferase subunit A (*PIGA)* gene resulting in a lack of glycosyl‐phosphatidylinositol (GPI) biosynthesis, a glycolipid that anchors more than 100 proteins at the cell surface, including CD55 and CD59. The CD55‐ and CD59‐deficient erythrocytes arising from *PIGA*‐mutated hematopoietic stem cells are targeted by complement. Activated complement results in intravascular hemolysis and hemoglobinuria which is enhanced by infections, stress, and often without any clear cause. Thrombosis, the major cause of mortality in PNH patients, is not fully understood but a least in part induced by high levels of free hemoglobin that consumes circulating nitric oxide, proinflammatory and prothrombotic cytokine release induced by activated complement, and other hemolysis‐independent mechanism of thrombosis [1]. Clonal selection and expansion of *PIGA*‐mutated hematopoietic stem cells could be the result of T and natural killer cell‐mediated autoimmune activation against modified GPI‐anchored surface antigens [2]. Taken together, the association between PNH and AA could result from common autoimmune activation occurring in the bone marrow. The C5‐inhibitory antibody eculizumab has shown a great ability to treat both PNH‐associated hemolysis and thrombosis.

Thymic abnormalities, hyperplasia or thymoma, are commonly associated with a broad spectrum of autoimmune diseases. MG is observed in up to 40% of thymoma cases and pure red cell aplasia is reported in up to 5% of thymomas as the most commonly associated autoimmune cytopenia [3]. These associations highlight the impact of thymic abnormalities on immune homeostasis and a role in promoting autoimmune disorders. In thymoma, neoplastic cells harbor immature features that lead to defective negative selection and have abnormal Human leukocyte antigen (HLA) expression with more autoreactive profiles [4]. Furthermore, impaired maturation and function of regulatory T cells (T_regs_) is described in both thymoma and thymic hyperplasia, especially in MG [5]. Importantly, thymectomy, commonly used as treatment for thymoma and MG, also promotes immunologic changes (involving T_reg_ function) [6] and could favor the development or progression of autoimmune diseases.

AA is observed in about 1% of thymoma and can occur long after thymoma diagnosis or thymectomy. Thymectomy usually does not control thymoma‐associated AA and a cyclosporine‐based therapy, with a reported response rate around two/third of the patients, has been proposed to be the gold standard in this rare situation. In the French cohort of AA associated with thymoma reported by Gendron et al., a minor PNH clone was found in up to 16.7% of cases [7] but, to our knowledge, only two cases of clinical PNH and thymic abnormalities have been reported. The first report described a patient who developed PNH 7 years after a diagnosis of thymoma with MG, treated by thymectomy. Interestingly, CD8^+^ T cells from the patient had direct cytotoxic activity against bone marrow progenitors, thereby potentially promoting clonal selection of *PIGA*‐mutated hematopoietic stem cells responsible for the PNH [8]. The second report described a patient with a thymoma‐associated AA that was treated by thymectomy, radiotherapy, antithymoglobulin, and cyclosporine and 3 years later developed PNH with intracardiac thrombus [9]. None of these PNHs were treated by eculizumab in their original report.

Our patient presented with association of nonsevere AA, PNH, and MG. Like the two other described cases, PNH developed years after thymectomy. Eculizumab was rapidly efficient in controlling hemolysis. Interestingly, spontaneous remission of AA occurred 6 months later, which still remains unclear since eculizumab has not shown any activity against the underlying immunological phenomena responsible for both AA and PNH. Spontaneous remission of AA has been described but these occurred mostly in the first 2 months and in infection‐ or medication‐induced AA [10]. In our case, it is probable that PNH clone further expanded and that the patient repopulated his bone marrow from his PNH clone. Importantly, the use of eculizumab appears to be safe in the setting of PNH and MG, with only one grade 3 infectious event despite long‐term use of steroids. Eculizumab has been recently reported to be effective in refractory AchR‐positive MG (REGAIN trial) [11], and thus may have contributed to maintain a relative stability of the MG in our patient (four flares in 4 years) despite infectious events and occasional PNH flares.

In conclusion, we report the first case of PNH associated with MG and safely treated by eculizumab. Although extremely rare, we believe that these conditions are not coincidental, since thymic abnormalities and thymectomy could be linked to autoimmune cytopenia, including AA, with the latter sharing a common pathophysiology with PNH.
